# Patient and health-care worker experiences of an HIV viral load intervention using SMS: A qualitative study

**DOI:** 10.1371/journal.pone.0215236

**Published:** 2019-04-11

**Authors:** Emilie Venables, Zibusiso Ndlovu, Dhodho Munyaradzi, Guillermo Martínez-Pérez, Elton Mbofana, Ponesai Nyika, Henry Chidawanyika, Daniela B. Garone, Helen Bygrave

**Affiliations:** 1 Médecins Sans Frontières, Southern Africa Medical Unit (SAMU), Cape Town, South Africa; 2 Division of Social and Behavioural Sciences, School of Public Health and Family Medicine, University of Cape Town, Cape Town, South Africa; 3 Department of Physiatrics and Nursing, Faculty of Health Sciences, University of Zaragoza, Zaragoza, Spain; 4 Médecins Sans Frontières, Harare, Zimbabwe; 5 Ministry of Health and Child Care (MoHCC), Harare, Zimbabwe; 6 Research Triangle Institute International, Harare, Zimbabwe; Centre for Sexual Health & HIV/AIDS Research, ZIMBABWE

## Abstract

**Background:**

Mobile Health or mHealth interventions, including Short Message Service (SMS), can help increase access to care, enhance the efficiency of health service delivery and improve diagnosis and treatment for HIV. Text messaging, or SMS, allows for the low cost transmission of information, and has been used to send appointment reminders, information about HIV counselling and treatment, messages to encourage adherence and information on nutrition and side-effects. HIV Viral Load (VL) monitoring is recommended by the WHO and has been progressively adopted in many settings. In Zimbabwe, implementation of VL is routine and has been rolled out with support of Médecins Sans Frontières (MSF) since 2012. An SMS intervention to assist with the management of VL results was introduced in two rural districts of Zimbabwe. After completion of the HIV VL testing at the National Microbiology Reference Laboratory in Harare, results were sent to health facilities via SMS. Consenting patients were also sent an SMS informing them that their viral load results were ready for collection at their nearest health facilities. No actual VL results were sent to patients.

**Methods:**

A qualitative study was conducted in seven health-care facilities using in-depth interviews (n = 32) and focus group discussions (n = 5) to explore patient and health-care worker experiences of the SMS intervention. Purposive sampling was used to select participants to ensure that male and female patients, as well as those with differing VL results and who lived differing distances from the clinics were included. Data were transcribed, translated from Shona into English, coded and thematically analysed using NVivo software.

**Results:**

The VL SMS intervention was considered acceptable to patients and health-care workers despite some challenges in implementation. The intervention was perceived by health-care workers as improving adherence and well-being of patients as well as improving the management of VL results at health facilities. However, there were some concerns from participants about the intervention, including challenges in understanding the purpose and language of the messages and patients coming to their health facility unnecessarily. Health-care workers were more concerned than patients about unintentional HIV disclosure relating to the content of the messages or phone-sharing.

**Conclusion:**

This was an innovative intervention in Zimbabwe, in which SMS was used to send VL results to health-care facilities, and notifications of the availability of VL results to patients. Interventions such as this have the potential to reduce unnecessary clinic visits and ensure patients with high VL results receive timely support, but they need to be properly explained, alongside routine counselling, for patients to fully benefit. The findings of this study also have potential policy implications, as if implemented well, such an SMS intervention has the potential to help patients adopt a more active role in the self-management of their HIV disease, become more aware of the importance of adherence and VL monitoring and seek follow-up at clinics when results are high.

## Introduction

Mobile Health, or mHealth, relates to the use of telecommunications infrastructure and uptake of mobile phones and other devices to support the provision of health services and help achieve global, community and individual level health targets [1]. By the end of 2014, mobile phone technology penetration was reported to be 90% worldwide [2]. In 2017, an estimated 85% of Zimbabweans per 100 inhabitants had mobile cellular subscriptions, suggesting that an mHealth intervention could be appropriate within this context [3].

There is growing evidence that mHealth interventions, including Short Message Service (SMS), can help increase access to care, enhance the efficiency of health service delivery and improve diagnosis, treatment and rehabilitation including supporting public health programmes for HIV and other conditions [1, 4–7]. Mobile phones have been used in the management of communicable diseases (including for patient tracing and partner notification for sexually transmitted infections), in health promotion programmes such as smoking cessation or vaccination campaigns and in capacity-building strategies [8].

Text messaging (SMS), compared to other communication means such as a traditional phone call or home visit, allows for low cost, instant transmission of information. SMS has been used to send people living with HIV reminders of their next appointments; information about ART refills; encouraging messages about adherence to ART and to advise them on nutrition and ART side effects [8]. SMS can also be used to transmit results of medical investigations such as screening tests or monitoring tests results for chronic conditions, and are not as invasive to people’s daily lives compared to receiving a phone call or a home visit from a health-care professional.

In 2016, UNAIDS reported that 36.7 million people were living with HIV worldwide, of whom 53% are receiving antiretroviral therapy (ART) [9]. Zimbabwe is one of the countries which is worst affected by the HIV epidemic in sub-Saharan Africa. Latest estimates reveal a national prevalence (15–49 years) of 13.3%. Approximately 1.3 million people are estimated to be living with HIV in Zimbabwe, with a reported 1,058,293 accessing ART by the end of December 2017 [10].

In 2013, the World Health Organization (WHO) recommended viral load (VL) monitoring for all patients on ART as the most accurate available measure of effective treatment response [11]. In Sub-Saharan Africa, as well as in other low and middle-income countries, VL testing is being progressively adopted by Ministries of Health as a cost-efficient test to monitor patients on ART [12]. In Zimbabwe, implementation of VL is routine and has been rolled out with support of Médecins Sans Frontières (MSF) since 2012 [13, 14]. As well as health benefits to individual patients, maintaining virological suppression has public health benefits including reducing the risks of HIV transmission [15]. Optimal VL suppression is defined by a VL <1000 copies/ml and second line ART initiation is guided by the presence of two consecutive high VL results (defined as ≥1000 copies/ml) [10].

Patients who fail to achieve VL suppression should receive enhanced adherence counselling (EAC) prior to repeat VL testing. At least two sessions of EAC are recommended after a high VL result, and if the VL remains high, assessment for second line treatment should take place. VL testing should be carried out annually, or every six months for those with a high VL result [10].

In many resource-limited countries with a high HIV burden, current HIV VL technologies are restricted to centralised laboratory testing, resulting in long turnaround times (TAT) for results which can in turn lead to the attrition of patients in the VL testing cascade. One identified example of this is mHealth services, which have already been proved acceptable in Malawi and Zambia to deliver early infant HIV diagnosis via SMS [16, 17]. To the best of our knowledge, SMS has not been used for similar purposes elsewhere in Zimbabwe.

This qualitative study explored the experience of using SMS to send information about the availability of VL results to patients in rural Zimbabwe, and explored patient and health-care worker (HCW) perceptions of the intervention.

## Methodology

This was a qualitative study, in which data was collected using in-depth interviews (IDIs) and focus group discussions (FGDs). The study was conducted during April 2015 in seven health centres across the districts of Gutu and Buhera in rural Zimbabwe.

### SMS intervention

**Fig 1:**
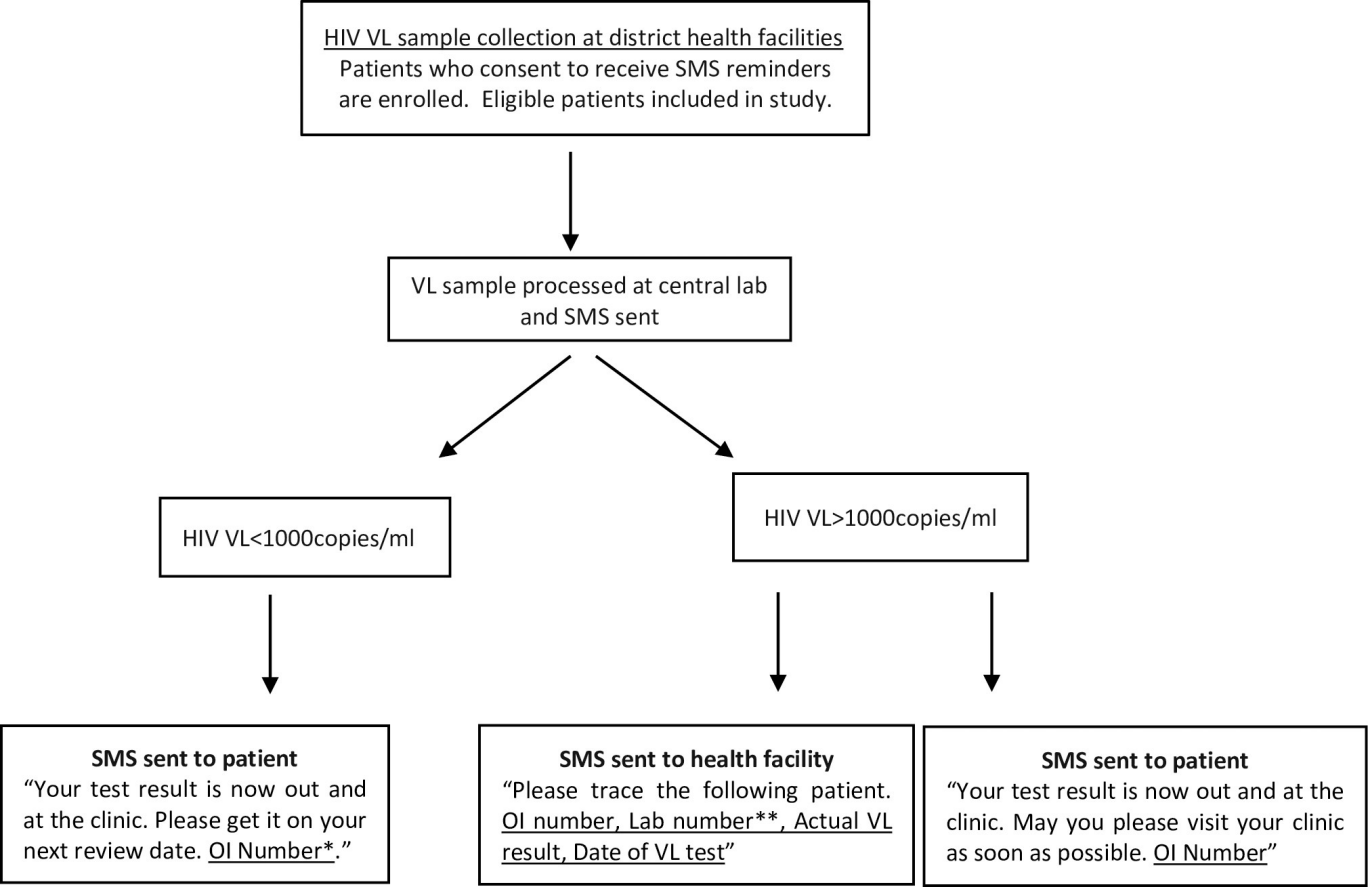
Summary of the mHealth programme. * OI number: unique patient ART identifier **Lab number: unique patient identifier in the laboratory.

From June 2014, as part of routine programmatic laboratory services, VL results from the centralised National Microbiology Reference Laboratory (NMRL) in Harare, the capital of Zimbabwe, were sent to a total of 21 health facilities in the two districts of Gutu and Buhera via SMS, along with the standard paper-based results (Fig 1). Combined, the 21 study facilities provide ART to approximately 70 percent of the patients on ART in the two districts. The intervention was intended to reduce the amount of time that patients waited for their VL results and in turn, reduce the amount of time that patients who needed enhanced adherence support had to wait for it. Preliminary results show that the median time from reporting of the VL result at the laboratory, to starting EAC reduced from 47 days (IQR 29–71) to 30 days (15.3–51.5) after the introduction of the intervention [18]. Moreover, the median TAT to EAC enrolment among patients self-reporting following receipt of an SMS was 13 days [IQR: 10–25].

All patients who consented at the time their test sample was taken also received an SMS relating to the result of their VL test. Messages told them that their results were ready to collect from their nurse and that they should visit the clinic immediately (VL ≥1000 copies/ml), or that they should wait until their next scheduled appointment if their VL result was <1000 copies/ml or undetectable. Patients were never sent messages with their actual VL results to avoid potential stigmatisation or problems that could arise with phone sharing.

The adult literacy rate in Zimbabwe is estimated to be 86.69%, thus for the purposes of implementing such an intervention for the first time in this context, it was decided that English should be used to send messages to patients and to health-care workers [19]. In addition, there are many different languages spoken within the study area, and English was selected as being common to all. Whilst Shona is one of the main languages spoken in the study sites, it was decided that the messages should first be piloted in English, before considering translation into other languages, for ease of sharing findings with other partners in other areas.

Patients gave their mobile phone number at the point of consent, after a verbal informed consent process in Shona in which the content of the messages was discussed and patients were asked to consider whether or not they would be comfortable to receive an SMS related to their VL, and were also made aware that their results would also be communicated between the laboratory and their clinic. All messages were designed and approved by the Ministry of Health and Child Care HIV Counselling Unit before implementation.

### Sampling and recruitment

Purposive sampling was used to invite patients and HCWs to participate in this qualitative study linked to the intervention described above. HIV positive patients on ART were eligible to participate if they were between 18 and 60 years old, had lived in the districts of Gutu or Buhera for a minimum of two years, had previously consented to participate in the SMS intervention and had received at least one text message in the previous two months. Patients were enrolled in the study by a clinic nurse or counsellor during routine consultations. The sampling framework was designed to ensure maximum variation, to ensure the inclusion of male and female patients, differing viral loads (≥1000 and <1000copies/ml), patients from different facilities and those living differing distances from where they accessed care to ensure that, as much as possible, participants in the qualitative study represented the wider intervention population. HCWs were selected on the basis of their experience and involvement in the intervention, as well as their role. We ensured a range of roles including nurses and counsellors were included in the study.

### Data collection and analysis

A total of four Focus Group Discussions (FGDs) with 21 patients and one FGD with nurses and counsellors were conducted, in addition to 10 key informant interviews with HCWs and 22 IDIs with patients. Each FGD had between five and six participants. All IDIs and FGDs were conducted in Shona using semi-structured guides (see S1 File) with open-ended questions, and were audio-recorded before being transcribed and translated into English. All interviews and FGDs took place in quiet, private rooms within the health facilities. FGDs and IDIs were conducted by a team of eight trained Zimbabwean, Shona-speaking researchers (five male and three female) and participants were invited to IDIs or FGDs several days before the data collection. Researchers were paired to the gender of the interviewees to elicit unrestricted discussions, as this was believed to be more culturally appropriate. Interviewers worked in pairs for interviews: one person took notes and observed non-verbal communication and the other asked questions. Each FGD was conducted by two or three researchers to ensure that accurate notes were taken and to ensure the full involvement of all participants. Interviews and FGDs were conducted until saturation was reached, in which no new findings were being revealed, and when study participants from the different groups and different facilities were sharing similar experiences and providing similar answers to questions. Each participant took part in either an FGD or an IDI, but not both.

Four researchers transcribed the audio-recordings before translation from Shona into English and verification by the research team. It was not possible to share transcripts with participants. Data were coded using inductive coding and thematically analysed by the principal investigator using NVivo software. Findings were then discussed amongst the team to identify and clarify any discrepancies during the coding and analysis process and ensure that findings were valid and that quality of analysis was assured. The five main themes presented below were chosen after discussion within the team based upon the coding of the transcripts. An initial 52 codes were developed, which were then grouped into the five main themes.

### Ethical considerations

The study was approved by the Médecins Sans Frontières Ethics Review Board Geneva, Switzerland (1503) and the Medical Research Council of Zimbabwe (MRCZ/A/1903). Participants gave their written informed consent before participating in interviews or FGDs, in addition to the verbal consent that they gave when originally agreeing to participate in the SMS intervention itself. The informed consent process was administered in Shona by a member of the research team who was not involved in the clinical or counselling follow-up of the patient. We have provided minimal demographic data in the results section to prevent participants from being recognised.

## Results

Of the 43 patients who participated in the study, 23 were female. The median distance from participants’ homes to their health facilities was 2km. Only 25.6% of the patient participants had a suppressed VL (<1000 copies/ml). The key informants consisted of nurses, counsellors and nurse aides. Most of the participants were employed and reported being married (90% and 85% respectively). Types of employment included farming, transport and fishing. Amongst patients who were interviewed, the length of time since HIV diagnosis ranged from six months to thirteen years, with a median of 66 months since diagnosis.

The main themes identified during analysis which are presented below are: patients’ understanding of the messages, perceived benefits of the intervention, unintended disclosure, the perceived effect on patient’s health-seeking behaviour and the acceptability of the message language and content.

### Patients’ understanding of messages

Most patients had only received one or two text messages at the time of the qualitative study, thus in some cases, had a limited understanding of the purpose of the intervention. HCWs believed that the messages were easy for patients to understand, and whilst patients knew that the SMS were from the clinic, the understanding of them varied with some participants having more of an understanding of the purpose of the messages and when to go to the clinic than others.

This FGD participant from Gutu found that the messages were easy to understand:

[T]here is a new system of SMS that lets you know the results are out or not. Instead of visiting the clinic after three months, you have an SMS to remind you that your results are out and you visit the clinic

A female patient from Mutero who was interviewed similarly stated that ‘*when you receive the SMS*, *it doesn’t show the results but tells you to visit the clinic as soon as possible’*.

However, another female patient with a high VL from Gutu described receiving an SMS but not understanding what it was for and if it was related to her HIV status:

I didn’t understand the meaning of the SMS, but after that they phoned me to tell me to visit the clinic but I was wondering if something had changed with my condition.

### Perceived benefits of text messages

HCWs were accepting of the intervention and believed it was beneficial to patients as well as making their own system of managing VL results more efficient. HCWs argued that as a result of the intervention they could fast-track those with high VL results for EAC, reduce the number of patients coming to the clinic unnecessarily and save patients time.

The HCW cited below found the SMS intervention advantageous because they believed that it enabled patients to come to the facility sooner, thus giving them the opportunity to access EAC earlier:

We see them in time and we will not be confused where to start with the patient. It improves adherence and their well-being. Patients come early and they are getting better. They are not having any problems.

Another HCW from Chimombe believed that the messages were encouraging patients to come to the clinic, even though some still ‘*rushed*’ unnecessarily:

They would come at the time the message directed them to come to the hospital, except the few who rush without reading the message.

Another counsellor believed that sending the text messages ‘*motivates’* patients and helps them to ‘*be more aware of the reason they are coming’*.

Perceived benefits from the perspective of patients included being able to know when their VL results were available without making unnecessary and lengthy clinic visits to the clinic to find out. One FGD participant liked the time-saving benefits of SMS because they believed ‘*you can cut certain procedures*, *like the lab*, *counselling and pharmacy’*. During an FGD in Gutu, participants also described the economic benefits of the SMS system, saying that ‘*it’s good because you don’t rush to see [your results]*, *because sometimes you face a problem of money’* and ‘*you can budget the money’*.

This nurse from Nerutanga considered the practical benefits for clinic staff, and believed that the messages prevented VL results from going missing and made the management of results easier:

The SMS comes earlier than the hard copy and it’s reliable because even if the hard copy gets lost along the way, we will be aware that this specific patient has a high viral load.

Another nurse from Rambanepasi Clinic who was interviewed echoed her words:

It’s helping us to help the patient, even when we have not received the hard copy. The programme has helped to improve the well-being of patients. We quickly find ways of assisting the patients with high viral loads instead of waiting for the hard copy. It helps us to help the patient before they deteriorate by conducting EAC, repeat viral loads and lab observations.

The benefits of receiving results by SMS rather than hard copy was also stressed by this nurse from Gutu, who stated that ‘*they can come even if the hard copy is yet to come…it has become easier and faster to get in touch with patients about their viral load’*. Her words were echoed by several health-care workers who felt that previously results could go missing or be marked as ‘pending’ for several months, delaying efforts to provide enhanced adherence counselling or switch patients to second line treatment if required.

### Unintended disclosure

Patients were asked about phone ownership during interviews: all participants owned a phone as this was a requirement of participating in the intervention, and of these, 58% of patient interviewees and 30% of patient FGD participants stated that they shared their phone with someone else. People reported sharing their phones with their husband or wife, their child or children. All participants stated that the person they shared their phone with knew their HIV status, but that they had not disclosed to everyone around them, such as family or friends. Whilst HCWs were concerned that messages relating to VL results could be read by people other than the patient, this was not raised as a serious concern by patients themselves. Three FGD participants in Gutu did not believe that phone-sharing would lead to unintended disclosure and mentioned that either they did not share their phone or that they had no concerns if their partner or a family member–including wives, husbands and children—answered it.

Sharing or discussing the content of the messages with other people, such as relatives, was dependent upon the individual patient and whom they had disclosed their HIV status to. Some individuals asked another person to translate the message and read it out to them if they were not confident reading English.

There were, however, some examples of patients such as this male FGD participant below who did not want the actual VL result to be mentioned in the SMS because he shared his phone with other people:

I’m a driver so when you are driving you can give [your phone to] someone to read the SMS. The SMS is good if it doesn’t specify the viral load. My SMS said ‘your results are out, you can visit the clinic on the next review date’. To receive the precise VL is not good.

### Health-seeking behaviour: Visiting the clinic after receiving a message

The time reportedly taken for the patient to arrive at the clinic after receiving an SMS varied between patients. HCWs reported that some patients came to the clinic immediately, even if it was not necessary; others came to the clinic earlier than their usual appointment and some did not attend the clinic for their appointment as expected. Overall, HCWs felt that patients came to the clinics earlier than they had done previously, but thought that ‘*some of the clients understand the instructions and others did not’*.

Some patients described feeling ‘*stressed*’ or ‘*anxious*’ upon receiving the SMS and one patient said that they ‘*ran*’ to the clinic because they thought there was a problem with their health and were concerned. One FGD participant from Gutu stated that ‘*I thought my results were not in a good condition*, *so I visited the clinic*’. Another female patient from the same clinic described feeling ‘*scared*’ when she received the SMS because she had been adherent to her treatment so thought there was a problem with her health:

I was scared because I was taking my pills properly, so why did my results say to go to the clinic as soon as possible?

However, there were also patients who highlighted that they appreciated the support of their clinic and they would prefer to speak to HCWs at their local facility rather than receive their actual VL results via text message.

Practical and logistical factors that caused delays in patients with a VL>1000 seeking health-care which could not be changed by the SMS intervention were also discussed by interviewees, such as long distances, employment or poor weather conditions. Another issue which was raised several times by health-care workers, which also affects the success of an intervention such as this one, was the reliability of the phone network, which one nurse pointed out was ‘*worsened by load shedding’*. Having access to a mobile phone, and one that was charged, was also cited by health-care workers as a potential challenge for the intervention as not everyone owned or could access a mobile phone or a reliable electricity supply. Shortage of transport was also highlighted by health-care workers as a challenge in reaching the clinic for those with high VL results who still needed to attend EAC.

### Acceptability of language and content of messages

Text messages were sent to patients in English, which created challenges for some recipients. HCWs and patients both suggested that messages should be written in the local language of Shona ‘*for those that don’t read English*’. One HCW explained that language was a barrier for some patients:

[N]o messages are delivered in vernacular languages so we encourage them to talk to relatives or children who understand English so that they help them.

HCWs were concerned that patients could misunderstand or misinterpret the messages if they were sent in English, but this was less frequently reported to be a challenge by patients.

Participants were asked whether they thought the words ‘viral load’ and the actual viral load results should be sent to individual patients, instead of the current message informing them that their results were ready. Several HCWs were again concerned about unintended disclosure through using the wording ‘*viral load’* in the messages, as they believed this would be understandable or recognisable to others. A counsellor in Gutu advised against using the actual VL results in numerical form as the ‘*patient may panic and may not understand the large figures’*. Patients had differing views about the content of the messages depending on their own personal preference: some stated a preference for the ‘*actual figure so that I will be calm before going to the clinic’* whereas others did not want to receive a number in case they became ‘*distressed’* or did not know how to interpret it.

## Discussion

mHealth interventions such as the one described above show how SMS is an acceptable form of communication for patients to receive information about the availability of their VL results. This study described an innovative SMS intervention from the perspective of HCWs implementing it and the patients who opted in to receive messages. The main perceived benefits of the intervention included being able to give patients information about their VL results without them having to come to the clinic, and ensuring that those who needed to come to the clinic because of a high VL did so more quickly. Implementation can be challenging, however, if infrastructure and clear responsibility for explaining the SMS system to patients and for acting on the information sent by SMS are not put in place. Challenges also occur if the patients do not fully understand the messages and the action they are required to take when receiving them, suggesting that similar interventions would need to focus more time explaining the intervention and its purpose.

Our results show that some patients did not completely understand the intervention, possibly due to assuming it was part of their routine care when they gave their informed consent to receive messages, or insufficient time being spent by the HCWs explaining the procedure prior to their viral load being taken. One potential solution for this would be for future interventions to send a test message to participants upon enrolment so that they can view the message, know what to expect and understand what to do when similar messages are sent to them in the future. Patient concerns about understanding their VL results and their concerns about their health may also have been linked to policy changes in Zimbabwe during the study period, in which CD4 testing for monitoring ART among stable patients was stopped in areas where VL testing was available, and VL testing was used instead. Increased advocacy is needed so that more patients know to ask for VL testing and their VL results, as well as how to interpret them, in turn increasing demand and acceptance of VL testing for HCWs and patients.

One of the objectives of this intervention was to reduce the number of patient visits made to the clinic and to reduce the time taken between the high VL result and commencing EAC. The reported time taken between receiving the SMS and coming to the clinic varied between patients and clinics and for this study, we were reliant on patient and HCW reports. A subset of this study was a retrospective quantitative analysis which showed that the time from reporting of the VL result to starting EAC reduced from 47 days (IQR 29–71) to 30 days (15.3–51.5) after the introduction of SMS for VL results [18].

In some cases, patients reportedly visited the clinic when they did not require EAC, but this suggests that they had a positive relationship with their HCWs that enabled them to attend the clinic outside of their scheduled times. As many patients had known their HIV status for several years, it is likely that they were familiar with the health-care system and their local clinic, thus findings may not be the same amongst groups of newly initiated patients. In the case of SMS interventions, ensuring people understand why they receive messages should help them to act appropriately. This would be even more important to consider if patients were to receive their actual VL result via text message, instead of a message to inform them that the results are available.

The ‘*stress*’ and ‘*anxiety*’ described by some patients upon receiving the SMS suggests that their health is still a concern, and that undue distress could be caused to patients by sending them messages without a thorough explanation. This may mean that many patients may still be struggling with adherence and HIV management despite receiving ongoing counselling and support from health-care workers, which then leads to concerns when contacted by the clinic.

Discussions about the use of mHealth in the field of HIV often focus on unintended disclosure. Many HIV-related mHealth studies from other contexts including China, South Africa and India report concerns about stigma and unintended disclosure relating to viewing messages meant for someone else but interestingly, this was not noted in this particular context in rural Zimbabwe [5, 20, 21]. Similar to a multi-cited study by Giguere et al. [22] where participants had ‘low security concerns’ relating to SMS in a rectal microbicide trial, our study shows that many prior assumptions about unintended disclosure did not apply in this context, as patients did not have a problem receiving messages about VL results on their phones. HCWs were more concerned about unintended disclosure than patients, showing that it is important to discuss issues of disclosure with facility staff, as they may in fact be more cautious than their patients. This also suggests that the HCWs in this study were very protective over their patients and concerned about their ability to understand the messages, when an intervention such as this one should be considered as a tool to empower patients. Again, the length of time since diagnosis or initiating treatment may affect disclosure, thus a cohort of newly initiated patients may yield different findings around disclosure to this study.

Despite the choice to send messages in English as this was the most common language across the different groups of people living within the study area, the language of the messages created some problems in comprehension, with one of the main recommendations from HCWs being to adapt messages into Shona for these particular clinics. This can be challenging in a context such as Zimbabwe, as well as in many other African settings, where several different languages may be spoken in the same region, but is useful to consider when implementing similar interventions elsewhere, and suggests the need to further pre-test messages before implementation. Our findings also suggest that patients have differing views on whether such messages should contain mention of VL or the actual VL results themselves: future interventions could consider further pre-testing of messages or, if possible, allowing patients a choice of whether or not they receive a message informing them that their results are ready or receive the actual results themselves.

Whilst some barriers to accessing health-care such as distance from the clinic, employment obligations, lack of money for travelling and bad weather causing flooding cannot be removed by the implementation of SMS, programmes such as the one described above can provide patients and HCWs with quicker VL results, and have the potential to reduce unnecessary visits to the clinic. In this case, mHealth can help improve VL services and provide support in a context where there are ongoing challenges impacting on access to health-care. There are also practical issues to consider for those involved in implementing mHealth interventions in rural areas where individual phone ownership, access and network challenges remain problematic.

HCW challenges relating to the management and ownership of the intervention, show that when the mobile phone used for sending messages to patients is shared this can lead to a lack of individual responsibility for specific tasks. This can, in turn, lead to poor tracing of patients or being unable to provide timeous follow-up or record VL results accurately. This could be improved by clearly identifying individuals who would be responsible for recording the VL results and messages during training. They did, however, appreciate the messages, as many had reported previous problems managing the hard copies of VL results which came to their facilities.

This study has several strengths, including providing the perspectives of patients and those implementing the intervention alike. The purposive sampling framework was designed to ensure maximum variation, and the participants in the study included those from different facilities, men and women, patients with high and low viral loads and living different distances from facilities. There are several limitations to this study. Firstly, most patients had only received one or two messages about their VL results since the start of the intervention, meaning that some of the challenges reported may have reduced over time and that these results are only preliminary. In addition, their responses may have been affected by recall bias. This study was conducted in a rural population and caution should be taken when generalising these results to urban HIV cohorts. As this was a qualitative study designed to understand people’s experiences of the intervention, it was not intended to measure impact.

## Conclusions

This study described a unique intervention in which SMS technology was used to send messages to patients about the availability of their VL results in a rural context in Zimbabwe. HCWs and patients found the intervention acceptable, and there were relatively few concerns from patients. More emphasis needs to be placed on explaining the purpose and content of messages, so that patients understand why they are going to be receiving messages, what the messages mean and what—if any—action should be taken. Messages such as those described above have the potential to empower patients through giving them more control over when they come to the clinic, as well as reducing congestion and waiting times in clinics, but SMS alone will not bring a drastic change in turnaround time for patients to start EAC.

The findings of this study also have potential policy implications, as if implemented well, such an SMS intervention could help patients adopt a more active role in the self-management of their HIV disease, become more aware of the importance of adherence and VL monitoring and seek follow-up at clinics when their results are high.

## Supporting information

S1 File interview guides in English and Shona. (DOCX)
