# Supplementary material for: Patient and health-care worker experiences of an HIV viral load intervention using SMS: A qualitative study
Source: PLoS One. 2019 Apr 11;14(4):e0215236. doi: 10.1371/journal.pone.0215236 (PMC6459516; doi:10.1371/journal.pone.0215236)
Supplement: S1 File — (DOCX) [file pone.0215236.s001.docx]

# English guide to in-depth interviews with patients

| **Code** | **Category** | **Information to be probed from participants** |
| --- | --- | --- |
| **Demographics** (To get from all IDI and FGD participants) | Age | Age of participant |
|  | Sex | Sex of participant |
|  | Occupation | Occupation/Income-generating activity |
|  | Education | Education/Level of literacy |
|  | Religion | Religion (specify church) |
|  | Family | Marital status/Household composition |
|  | Disclosure | Partner/Relatives aware of HIV status? |
|  | Distance to facility | How much time does it take you to arrive to the health facility from your home? |
| **Mobile Phone** | Ownership | Do you own a mobile phone? |
|  | Sharing | Do you share your mobile phone? With whom? |
|  |  | Does that person have access to your SMS? |
|  |  | Is he/she aware of your HIV status? |
|  | Phone usage | Knowledgeable with functions of receiving/sending SMS? |
| **HIV Management** | HIV Test | Since when are you aware of your HIV status? |
|  | ART Initiation | When were you started on ART? |
|  | Adherence to ART | On average, how many days per month do you miss taking your ART? |
|  |  | What explanation were you given on the consequences of not taking your drugs? |
|  | Follow-up | What do the nurses currently do to follow up that your HIV treatment is working? |
|  | Lab Results | Can you explain what a VL test means? |
|  |  | Do you remember your last VL result? |
|  |  | Ideally, below what number would you like your VL test result to be? |
|  | Healthcare measures | If your VL were/is <1000, what are the healthcare measures you have to comply with? |
|  |  | If your VL were/is above 1000 copies, what are the healthcare measures you have to comply with to get your VL <1000 copies/ml ? |
| **SMS Program** | Enrolment SMS | Who asked you for permission to send an SMS on your VL result when they took your blood? |
|  |  | What exactly was explained to you about the SMS you were to receive? |
|  | Reception SMS | Can you estimate how many VL SMS reminders have you ever received? |
|  |  | When was the last time you received your VL result in your phone? |
|  |  | Do you remember what the SMS said? |
|  |  | What does that mean? |
|  |  | What did you do when you received the SMS? |
|  |  | Have you ever received any encrypted/un-readable SMS from the program? |
|  | Sharing message | Did you communicate this SMS with anybody? With whom? |
|  |  | What and how did you communicate that SMS to that person? |
|  |  | Why did you communicate it (e.g. share information, did not understand/remember content of message…)? |
|  |  | Which feedback did you hear from that person? |
| **Healthcare-seeking behaviour** | Visit to Clinic for patients with VL ≥1000 copies/ml | If the SMS said that you should go to the clinic, how long did it take for you to reach the clinic? |
|  |  | If you did not do as instructed (go to clinic), why not? |
|  |  | If you travelled to the clinic, please explain the whole process that you undergo (whom do you see first with your SMS message, do you queue with all other patients or you are prioritized, how long will it take till you are served?) |
|  |  | Did you request that person to explain you the message again or was it clear? |
|  |  | Did you request the counsellor/nurse to tell you the exact result of your VL test? Did you get the exact result? |
|  |  | Did the message/result communicated to you by the nurse or counsellor match with the SMS you received? |
|  | Visit to clinic for patients w/VL <1000 copies/ml | If the SMS said that you should not go to the clinic, would you have gone anyway to hear the result from your nurse? |
| **Perception** | Adherence (to both type of patients) | Do you consider your motivation to adhere to treatment got a boost receiving this message by SMS, or nothing changed? |
|  | Anxiety | Did you feel anxious while waiting to receive the SMS? More or less anxious than if you had to return to the clinic to receive your result from the nurse? |
|  |  | In a 1-10 scale, how would you rate your level of anxiety before receiving the SMS? And after? |
|  |  | Can you describe your feelings when you received the SMS asking you to return to the clinic? **(for patients with VL ≥1000 copies/ml )**  **OR**  Can you describe your feelings when you received the SMS asking you NOT to return to the clinic? **(for patients with VL below 1000)** |
|  |  | What was your first thought? |
|  |  | Would you feel OK if the program would only send msg in case of BAD results to safe cost? If no, why not? If yes, why? |
| **Safety** | Interpretation | Was it difficult for you to identify what the message was about? Why? |
|  |  | If it took time, was it because you had forgotten the nurse´s instructions? Or because no instructions were given to you or other reasons? |
|  |  | Did you feel insecure about the content of the message or had to ask for advice from any other person in your household? |
|  | Adequacy | Is the content of the message appropriate for you? Why? |
|  |  | How would you change the message if you consider it inappropriate? |
|  | Harm/  Disclosure | Were you afraid someone else could read the message and find out you were HIV positive? |
|  |  | If anyone unaware of your status had read the message, what would you have said not to reveal its real meaning? |
|  | Trust | Did you trust the content of the message? Why? |
|  |  | Have you ever considered the message might have been for a different person? |
| **Satisfaction** | Benefits | Was getting your result by SMS an easier and more acceptable system than when you had to return to the clinic for the result? Why? |
|  |  | Can you describe the advantages of receiving SMS? |
|  |  | Did you receive the message in a timely manner (before your next appointment at the health centre)? |
|  |  | Are you comfortable with not travelling to the clinic to get your results if these are fine? |
|  |  | Does receiving an SMS asking you not to return to the clinic help you to save transportation costs? |
|  |  | Does receiving an SMS help you to prevent having to stop looking after your business/going to work/farming…? |
|  |  | Does receiving an SMS help you to prevent stopping taking care of your children/elders? |
|  | Nurses´ Role | Are you satisfied with the explanations on the SMS service you received from the nurses/counsellors? Why? |
|  |  | What information on the SMS service would you have liked to receive from the nurse/counsellors? |
|  | Overall Satisfaction | In general, are you satisfied with this SMS service? Why? |
|  |  | Do you want to continue receiving these messages? |
|  |  | Would you recommend the service to other HIV-positive people? |
|  |  | What would you suggest to improve the service? |
|  | Way Forward | Would you rather receive the precise VL result besides the instruction on going back or not to the clinic?  Would you know how to interpret an actual result?  Would you accept the word ’viral load’ to appear in the message? |
|  |  | If you were a HIV positive mother awaiting the result on your child HIV status; would you accept the actual result to come by SMS?  For less stigmatised diseases (such as diabetes) do you think laboratory results could be send by SMS? What other diseases do you think laboratory results could be sent by SMS? |

#

# Shona guide to in-depth interviews with patients

| **Code** | **Category** | **Information to be probed from participants** |
| --- | --- | --- |
| **Demographics** (To get from all IDI and FGD participants) | Age | Makore ekuberekwa |
|  | Sex | Murume kana mukadzi |
|  | Occupation | Basa ramunoita rinokupa mari |
|  | Education | Makadzidza kusvika papi? |
|  | Religion | Sangano ramunonamata muri |
|  | Family | Makaroora kana kuroorwa here? Muri vangani mumhuri? |
|  | Disclosure | Partner/Relatives aware of HIV status? |
|  | Distance to facility | Munotora nguva yakadii kufamba kubva kumba kwenyu kusvika kuchipatara? |
| **Mobile Phone** | Ownership | Mune foni here |
|  | Sharing | Pane anomboshandisa foni yenyu here? Ndiani wenyu? |
|  |  | Anomboshandisa phone yenyu anokwanisa kuona mamessage enyu here? |
|  |  | Anoziva pamakamira here maringe ne HIV? |
|  | Phone usage | Munoziva here kutumira kana kugamuchira meseji? |
| **HIV Management** | HIV Test | Makaziva mamiriro enyu maringe ne HIV riini? |
|  | ART Initiation | Makatanga kumwa maARV riini? |
|  | Adherence to ART | Munodarikira mazuva mangani kumwa maARV enyu pamwedzi |
|  |  | Tsanangudzo yamakapiwa maringe nezvinokwanisa kuitika mukasamwa maARVs enyu ndeyekuti chii? |
|  | Follow-up | Vanamukoti vari kuitei kwamuri kuti vaone kana maARV ari kushanda mamuri? |
|  | Lab Results | Nditsanagurireiwo kuti kuongororwa VL zvinorevei? |
|  |  | Muchiri kurangarira here mamirire eVL yamakapedzisira kutorwa? |
|  |  | Mungada kuti viral load yenyu igare iri pasi penumber ipi? |
|  | Healthcare measures | Toti viral load yenyu iri pasi pe 1000 ndezvipi maringe neutano hwenyu zvamungaita kuti igare yakadaro? |
|  |  | Dai viral load yenyu iri pamusoro pe 1000 ndezvipi zvamungaita kuti idzike kusvika pasi pe 1000? |
| **SMS Program** | Enrolment SMS | Ndiani akakukumbirai mvumo yekutumirwa meseji maringe ne viral load yenyu pamakatorwa ropa? |
|  |  | Chii chakanyatsotsanangurwa kwamuri maringe nemeseji yamaifanira kuzogamuchira? |
|  | Reception SMS | Mungave nefungidziro yekuti kusvika pari nhasi mawana mameseji mangani eViral Load? |
|  |  | Makapedzisira kugamuchira meseji yemaresults eviral load mufoni menyu riini? |
|  |  | Muchiri kurangarira kutiyakange yakanzi kudii here? |
|  |  | Zvinorevei zvamataura? |
|  |  | Chii chamakaita pamaka gamuchira meseji iyi ? |
|  |  | Mati mambowana meseji isingaverengeki here ine chekuita nechirongwa ichi? |
|  | Sharing message | Pane wamakaudza here nezvemeseji iyi? Ndiani? |
|  |  | Makamuudza kuti chii uye sei nemeseji iyoyo? |
|  |  | Nemhaka yei makamuudza (muenzaniso…kungotaurwo kuti azive, ndakange ndisinga nzwisisi/rangariri zvaiva mumeseji yacho ) |
|  |  | Akakupindurai kuti chii munhu iyeye? |
| **Healthcare Seeking Behaviour** | Visit to Clinic for patients with VL ≥1000 copies/ml | Kana meseji yakati dzokerai kukiriniki, zvakakutorerai nguva yakadii kudzokera? |
|  |  | Kana musina kuita zvakatsanangurwa nemeseji, makaregerei? |
|  |  | Kana makadzokera kukiriki, tsanangurai zvose zvakaitika (ndiani wamakatanga kuona, nemessage yenyu, munotevedza mutsetse here kana kuti munonzi mupfuurire mberi kwevamwe varwere, zvinokutorerai nguva yakareba sei kuti mubatsirwe). |
|  |  | Makakumbira kutsanangurirwa zvakare here kuti inorevei message kana kuti zvanga zvakakujekerai? |
|  |  | Makakumbira mukoti kana kuti chipanga mazano result yenyu chaiyo here? Makaudzwa maresults chaiwo here? |
|  |  | Messeji kana kuti result yakataurwa namukoti kana chipanga mazano yaienderana here nemessage yamakagamuchira? |
|  | Visit to clinic for patients with VL <1000 copies/ml | Kana meseji yaiti musaenda kukiriniki, mungadai makangoenda here kunonzwa kubva kuna nurse? |
| **Perception** | Adherence (to both type of patients) | Mungati here meseji yakaku kurudzirai kuti mutevedzere nguva yekumwa mapiritsi enyu kana kuti hapana chakashanduka? |
|  | Anxiety | Makanzwa kusagadzikana here kana kugadzikana kwakadii makamirira meseji? |
|  |  | Pachiero chekubva pana 1 kusvika 10 mungati kusagadzikana kwenyu kwaive papi musati magamuchira meseji uye nepamakazogamuchira meseji? |
|  |  | Mungatsanangura here zvamakanzwa pamakagamuchira meseji ichiti mudzokere kukiriniki?  **kana**  Mungatsanangura here zvamakanzwa pamakagamuchira meseji ichiti musadzokere kukiriniki? |
|  |  | Pakutanga makafunga chii? |
|  |  | Zvakakunakirai here kuti chirongwa ichi chiitirwe apo maresults anenge asina kunaka chete kuitira kudzikisa kudhura kwekutumira meseji? Kana zvisingakuitirii sei muchidaro? Kana zvichikuitira, sei muchidaro? |
| **Safety** | Interpretation | Zvakakunetsai here kuti muzive kuti meseji yabva kupi uye yaiva yechii? Sei muchidaro? |
|  |  | Kana zvakatora nguva imhaka yekuti mange makanganwa zvakataurwa namukoti here?  Kana kuti hamuna kunge matsanangurirwa nezvazvo kana chimwe chikonzero? |
|  |  | Makanzwa kusagadzikana here nemhaka yezvaitaura meseji kana kuti makakumbira rubatsiro kubva kune mumwe munhu ari mumba menyu? |
|  | Adequacy | Zviri mumeseji munoona zvakakodzera here? Sei muchidaro? |
|  |  | Mungashandura sei mesejie iyi kana muchiti haikodzeri? |
|  | Harm/ Disclosure | Maitya here kuti mumwe munhu angaverenga message akaziva kuti muri kurarama neutachiwana hweHIV? |
|  |  | Dai mumwe munhu asingazivi mamiriro enyu maringe neutachiwana hwe HIV akaverenga meseji yenyu, maiita sei kuti asaziva zvainoreva? |
|  | Trust | Makavimba here nezvaiva mumeseji iyi? |
|  |  | Makafunga here kuti message iyi yaiva yeumwe munhu asiri imimi? |
| **Satisfaction** | Benefits | Kugamuchira kwamakaita meseji makaona kuri nyore here uye kuchigamuchirika kudarika dai makadzokera kukiriniki here? Sei muchidaro? |
|  |  | Mungatsanangura here zvakanakira kugamuchira meseji? |
|  |  | Makawana meseji paine nguva here (Zuva renyu rekudzokera kuchipatara risati rasvika)? |
|  |  | Makasununguka here nekusadzokera kukiriniki kuti munoudzwa maresults enyu kana akanaka? |
|  |  | Kugamuchira meseji yekuti musadzokera kuchipatara kunokubatsirai here kuti muchengetedze mari yekubhadhara chekufambisa ? |
|  |  | Kugamuchira meseji yekuti musadzokera kuchipatara kunokubatsirai kuti musatadza kuita bhizimusi renyu, kuenda kubasa kana kurima……? |
|  |  | Kugamuchira message yekuti musadzokera kuchipatara kunokubatsirai here kuti muvane nguva yekuchengeta vana venyu kana vasharukwa…? |
|  | Nurses´ Role | Makagutsikana here netsanangudzo yakapihwa namukoti kana chipanga mazano maringe nechirongwa chekugamuchira meseji eVL? Sei muchidaro? |
|  |  | Ndeipi imwe tsanangudzo pamusoro pechirongwa ichi yamungati maida kunge makagamuchira kubva kuna mukoti kana chipanga mazano? |
|  | Overall Satisfaction | Muchitarisa zvese, munogutsikana here nechirongwa chemameseji ichi? Sei muchidaro? |
|  |  | Munoda here kuramba muchigamuchira mameseji aya? |
|  |  | Mungakurudzira vamwe vanhu here vanorarama neutachiwana hweHIV kuti vapindewo muchirongwa ichi? |
|  |  | Ndezvipi zvamungada kuti zviitwe kuti chirongwa ichi chiwedzere kunaka? |
|  | Way Forward | Mungada here kugamuchira maresults chaiwo pane kungoudzwa kuti mudzokere kana kusadzokera kukiriniki? Mungakwanisa kududzira maresults chaiwo?  Mungagamuchira here mashoko ekuti ‘viral load’ ave mumeseji? |
|  |  | Dai muri amai/baba vari kurarama neutachiwana hweHIV makamirira maresults eHIV emwana wenyu, mungagamuchira here result kuti iuye nemeseji?  Pazvirwere zvisina kushoreka kwakanyanya (zvakafanana neshuga) munofunga kuti maresults kubva kulab angatumirwe nemeseji here? Ndezvipi zvimwe zvirwere zvamunofunga zvingaita kuti maresults emuLab atumidzirwe semeseji pafoni? |
